# Lynch Syndrome-associated Mutations in *MSH2* Alter DNA Repair and Checkpoint Response Functions *In Vivo*

**DOI:** 10.1002/humu.21333

**Published:** 2010-10

**Authors:** Adam S Mastrocola, Christopher D Heinen

**Affiliations:** Neag Comprehensive Cancer Center and Center for Molecular Medicine, University of Connecticut Health CenterFarmington, Connecticut 06030-3101, USA

**Keywords:** Lynch Syndrome, DNA mismatch repair, missense mutations, DNA checkpoint response, *MSH2*, *MSH6*

## Abstract

The DNA mismatch repair (MMR) pathway is essential in maintaining genomic stability through its role in DNA repair and the checkpoint response. Loss of DNA MMR underlies the hereditary cancer disease Lynch Syndrome (LS). Germline mutations in *MSH2* account for approximately 40% of LS patients and of these, 18% are missense variants. One important clinical challenge has been discriminating between missense variants that are pathogenic and those that are not. Current analysis of missense mutations in *MSH2* is performed using a combination of clinical, biochemical, and functional data; however, suitable cell culture models to test the various functions of the DNA MMR proteins are lacking. Here, we have generated human cell lines stably expressing a subset of MSH2 missense mutants and tested their effect on DNA repair and checkpoint response functions. We have expanded on previous biochemical and functional analyses performed in non-human systems to further understand defects conferred by this subset of single amino acid alterations. The functional characterization of MSH2 missense mutants combined with clinical and biochemical data is essential for appropriate patient management and genetic counseling decisions. ©2010 Wiley-Liss, Inc.

## INTRODUCTION

The repair of single DNA nucleotide mismatches by the DNA mismatch repair (MMR) pathway is essential in maintaining genomic stability (Hsieh and Yamane, 2008; Li, 2008). Recognition of DNA basepair mismatches

occurs by a heterodimer of two MutS homologs, MSH2 and MSH6. Models of MMR mechanism propose that MSH2-MSH6 heterodimers load onto DNA via a mismatch and then recruit downstream MMR factors including a heterodimer of the MutL homologues MLH1 and PMS2 (Acharya, et al., 2003; Constantin, et al., 2005; Zhang, et al., 2005). In addition to their role in the repair of replication errors, these MMR proteins are required for the activation of cell cycle checkpoints and apoptosis in response to DNA alkylation damage (Stojic, et al., 2004a). The requirement of MMR was initially characterized in MMR-deficient strains of *E.coli*, which displayed tolerance to the DNA alkylating agent N-methyl-N′-nitro-N-nitrosoguanidine (MNNG) (Karran and Marinus, 1982). These observations were then extended to MMR-proficient human cell lines that were demonstrated to be 100-fold more sensitive to treatment with MNNG than MMR-deficient cells (Karran, 2001). Activation of the DNA checkpoint response to low-doses of MNNG is mediated through the ATR-Chk1 pathway, which induces a G_2_ cell cycle arrest and subsequent apoptosis (Stojic, et al., 2004b).

Inherited mutations in genes of the MMR pathway give rise to the autosomal dominant disorder Lynch Syndrome (LS; MIM# 120435), which accounts for 3-5% of all colorectal cancers. LS patients have a 60-80% chance of developing colorectal cancer in their lifetimes in addition to certain extracolonic cancers (Lynch, et al., 2009). The majority of LS patients carry a mutation in either *MSH2* (MIM# 609309) or *MLH1* (MIM# 120436), resulting primarily in a truncated, nonfunctional protein (Peltomaki and Vasen, 1997). However, of the mutations identified in *MSH2*, approximately 18% are missense mutations (Ou, et al., 2008). These single amino acid alterations are potentially powerful biological tools for understanding the mechanisms driving tumorigenesis in LS patients. We have previously investigated the *in vitro* biochemical defects associated with a subset of LS-associated missense mutations in MSH2 using purified recombinant proteins (Heinen, et al., 2002). All but one of the missense mutant proteins examined conferred significant defects on MSH biochemical activity. However, these data do not directly address the impact of these mutations on MMR functions in the cell. To date there has been limited data describing the contribution of *MSH2* missense mutations on MMR functions *in vivo.* In this study we extend our previous work on four missense mutations in *MSH2* to determine the consequences of their biochemical defects on *in vivo* repair and checkpoint response functions.

## MATERIALS AND METHODS

### Cell Culture and production of lentiviral cell lines

Hec59 cells [kindly provided by Drs. Thomas Kunkel and Alan Clark (Watanabe, et al., 2000)] were grown in DMEM:Nutrient Mixture F-12 (DMEM/F-12), containing 10% fetal bovine serum (FBS; Gibco). cDNA from wild-type or missense *MSH2* (GenBankNM_000251.1), including c.499G>C (p.Asp167His; D167H), c.1178A>T (p.Lys393Met; K393M), C.1571G>C (p.Arg524Pro; R524P), and c.1865C>T (p.Pro622Leu; P622L) generated previously (Heinen, et al., 2002), was PCR amplified and subcloned into the pCDH-EF1-MCS-T2A-Puro lentiviral expression vector (System Bioscience). All variant numbering is based on *MSH2* cDNA sequence and uses the A of the ATG translation initiation start site as nucleotide +1. Following lentiviral transduction, puromycin was added to Hec59 cells at a final concentration of 0.5 μg/ml. Bulk-infected cell populations were kept under puromycin selection until harvest. Analyses were performed from two separate infections.

### Chemicals, Antibodies, and Reagents

N-Methyl-N′-nitro-N-nitrosoguanidine (MNNG) was obtained from the National Cancer Institute Chemical Carcinogen Reference Standard Repository; CAS: 70-25-7. O^6^-Benzylguanine (O^6^-BG) and hydroxyurea were purchased from Sigma. Antibodies against PCNA (SC-56), MSH6 (SC-10798) and MSH3 (SC-11441) (for Western blot analyses) were purchased from Santa Cruz Biotechnology; MLH1 (550838 for Western blot analysis), MSH2 (556349 for Western blot analysis), MSH6 (610918 for immunoprecipitation) from BD Biosciences; Actin (A5060) from Sigma; Histone 3 (10799) from Abcam.

### Cell synchronization, drug treatment, and chromatin immunoprecipitation

Synchronization at the G_1_/S boundary was performed by treatment with hydroxyurea (HU). Cells were grown for 16 h in complete medium containing 2 mM HU, after which 25 μM O^6^-BG was added until the cells were released into serum free medium containing 5 μM MNNG and 25 μM O^6^-BG. After 1 h, cells were incubated in complete medium until harvest. Chromatin immunoprecipitation (ChIP) was performed as previously described (Mastrocola and Heinen, 2010).

### Cell Cycle and Survival Analyses

Cell cycle analyses were performed using propidium iodide (PI) staining for DNA content with a FACS Calibur flow cytometer (BD Biosciences) and was analyzed by Modfit (Verity Software House). Time points were performed in duplicate. For survival assays, 500 cells were plated then treated with 25 μM O^6^-BG for 2 h followed by 0, 0.5, 2, 5, or 10 μM MNNG and 25 μM O^6^-BG for one hour. Cells were incubated in normal growth media for 9 days. Subsequently, cells were harvested, fixed with methanol, and stained with crystal violet. The experiment was done in quadruplicate for each lentiviral cell line and time point.

### MM R Assay

The heteroduplex MMR substrate was prepared according to Zhou et al. with modifications (Zhou, et al., 2009). The p111 and p189 plasmids were a kind gift from Dr. LuZhe Sun. p189 encodes for a premature stop codon in the EGFP gene. To generate single-stranded (ss) DNA, XL-1 blue cells were transformed with p111. Subsequently, cells were infected with the M13KO7 helper phage (New England Biolabs) to generate viral particles, which were recovered by polyethylenglycol 8000 precipitation. Phage particles were banded by CsCl ultracentrifugation and the ssDNA was isolated by phenol-chloroform extraction. The MMR substrate was prepared by annealing the ssDNA circle to linearized, denatured DNA from p189. Excess ssDNA was removed by BND-cellulose. Excess dsDNA was removed by treatment with ExoV (USB). To assess MMR activity, cell lines were transfected with 1.5 μg of the heteroduplex plasmid and 1 μg of red fluorescent protein (RFP) expressing plasmid pDsRed2-N1 (Clonetech) using lipofectamine 2000. After incubation for 40 h the cells were harvested and analyzed for fluorescence intensity with a FACS LSRII-B flow cytometer (BD Biosciences) using BD FACSDiva software. GFP fluorescence was normalized to RFP expression to correct for differences in transfection efficiency.

## RESULTS

### Introduction and expression of MSH2 missense mutants in the MSH2-deficient cell line Hec59

**Figure 1:**
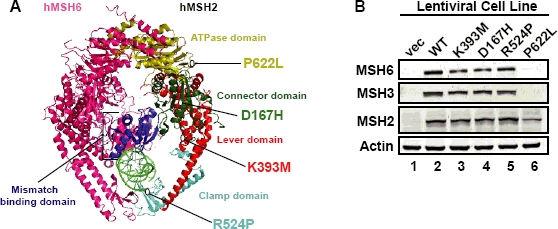
Structural location and expression of *MSH2* missense mutations. (A) MSH2 missense mutations mapped to the human MSH2-MSH6 crystal structure (Warren, et al., 2007). MSH6 is in pink, the subdomains of MSH2 are as indicated and mispaired DNA is lime green. (B) Whole cell extracts from Hec59 cells transduced with various lentiviral constructs were analyzed by Western blotting with antibodies against MSH2, MSH6, MSH3 or Actin as a loading control.

The goal of this study is to further understand the functional consequences of missense mutations in *MSH2* on MMR functions *in vivo*. We chose to examine four missense mutations: D167H, K393M, R524P, and P622L (Figure 1A), which were identified in four different LS patients with microsatellite unstable tumors (Heinen, et al., 2002; Leach, et al., 1993; Moslein, et al., 1996; Orth, et al., 1994). Stable Hec59 cell lines expressing MSH2 missense mutant protein were derived using a lentiviral system that confers both MSH2 expression and resistance to puromycin (Donnelly, et al., 2001). Hec59 cells are an endometrial cancer cell line that carries biallelic mutations in *MSH2* resulting in loss of a detectable protein product by Western blot (Figure 1B, lane 1). Bulk populations of infected cells were kept under puromycin selection and protein expression from each cell population was determined by Western blot analysis (Figure 1B). MSH2 protein expression was found to be similar between the wild-type and mutant expressing cell lines, except for MSH2(P622L), which was ∼50% of MSH2(WT). In Hec59 cells the MSH6 and MSH3 proteins are also not detectable by Western blot as their stability depends on formation of a heterodimer with MSH2 (Marra, et al., 1998) (Figure 1B, lane 1), however, introduction of wild-type (WT) MSH2 restored both MSH6 and MSH3 stability. MSH2(D167H), MSH2(K393M), and MSH2(R524P) were also able to restore MSH6 and MSH3 protein stability while MSH2(P622L) did not (Figure 1B).

### LS-associated missense mutations affect chromatin localization and MMR protein complex formation

**Figure 2:**
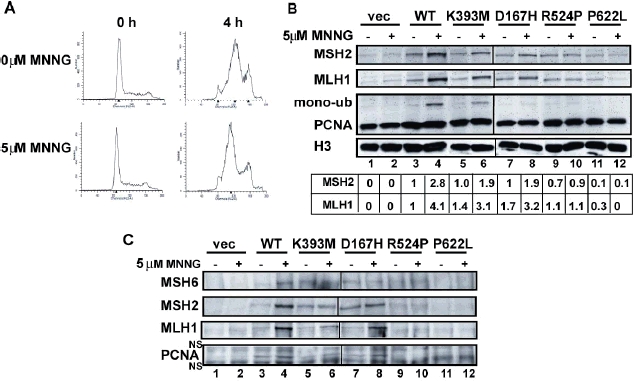
Chromatin localization and MMR protein complex formation of MSH2 missense mutants. Hec59 cells were synchronized with hydroxyurea (HU), treated with O^6^-BG and 5 μM MNNG or DMSO for 1 h then returned to normal growth media. (A) Cell cycle progression was monitored by flow cytometry. (B) Chromatin-enriched extracts prepared from MNNG treated or control cells at 4h post-HU release subjected to analysis by SDS-PAGE and Western blotting using antibodies against MSH2, MLH1, and PCNA. H3 was used as a loading control. MSH2 and MLH1 levels were quantified from at least three separate experiments and are reported as the average fold change over the MSH2(WT) levels in the absence of MNNG. (C) Chromatin-enriched extracts were subjected to ChIP analysis with antibody against MSH6 and interactions with MSH2, MLH1 and PCNA were probed by Western blot. NS refers to non-specific bands on the PCNA blot caused by the secondary antibody.

The recruitment and interaction of MMR proteins on the chromatin is an important step in the DNA MMR pathway *in vivo*. We have previously shown that MSH2 and MLH1 rapidly accumulate and form a protein complex with PCNA on the chromatin following treatment with MNNG (Mastrocola and Heinen, 2010). DNA MMR activity is most abundant during S-phase (Schroering, et al., 2007), therefore, to monitor chromatin localization we first synchronized MSH2(WT) Hec59 cells to the G_1_/S border with hydroxyurea (HU), a ribonucleotide reductase inhibitor. We observed the majority of both DMSO and MNNG-treated cells in the middle of S-phase at 4 h post-HU release (Figure 2A). To monitor the localization of MMR proteins to the chromatin, chromatin-enriched fractions were prepared from the infected cell populations at 4h post-HU release. The parental Hec59 cells lack detectable MSH2 protein (Figure 2B, lane 1 and 2). The faint band displayed in the vector only chromatin-enriched fraction is nonspecific and is distinguishable from MSH2 under different SDS-PAGE conditions (data not shown). A sub-population of wild-type MSH2 localizes to the chromatin during S-phase and accumulates to an almost 3-fold greater extent upon treatment with MNNG (Figure 2B compare lanes 3 and 4), whereas the MSH2(D167H) and MSH2(K393M) protein also localize to damaged chromatin to a slightly reduced extent (Figure 2B, lanes 6, 8). Interestingly, MSH2(R524P) chromatin-bound levels were similar to the MSH2(WT) during an unperturbed cell cycle; however, MSH2(R524P) failed to accumulate after treatment with MNNG (Figure 2B compare lanes 4 and 10). In contrast, MSH2(P622L) failed to localize to the chromatin in damaged or undamaged cells (Figure 2B compare lanes 4 and 12).

Reintroduction of MSH2(WT), MSH2(D167H) and MSH2(K393M) into Hec59 cells dramatically increased the chromatin-localization of MLH1 (Figure 2B), which is consistent with reports that MLH1 recruitment is MSH2-dependent (Hidaka, et al., 2005; Mastrocola and Heinen, 2010). As expected, there were significantly reduced levels of chromatin-associated MLH1 in MSH2(R524P) and MSH2(P622L) cell populations. Although we did not observe a significant difference in chromatin-associated PCNA levels in the MSH2(WT) cells after treatment with MNNG, we did observe a higher-migrating band on the PCNA blot that was significantly enhanced by DNA damage and may be a mono-ubiquitinated (ub) form of PCNA (Szüts, et al., 2005). We observed a similar increase in mono-ub PCNA in MSH(K393M) cells, but no increase for MSH2(R524P) and MSH2(P622L) cells populations. Interestingly, although MSH2(D 167H) accumulated on damaged chromatin and was able to recruit MLH1, the levels of mono-ub PCNA were significantly diminished compared to the MSH2(WT) (Figure 2B compare lanes 4 and 8).

To test whether our subset of mutants are able to interact with MLH1 and PCNA *in vivo*, chromatin-enriched extracts were prepared and then subjected to immunoprecipitation with antibody against MSH6. After treatment of MSH2(WT), MSH2(D167H) and MSH2(K393M) cells with MNNG, we observed the formation of a MMR complex containing MLH1, MSH2, MSH6, and PCNA (Figure 2C). In contrast, MMR protein complex formation was not detectable in the MSH2(R524P) cell line, which is consistent with the inability of this mutant to localize to the chromatin. The inability of MSH2(P622L) to interact with MLH1 and PCNA is most likely due to a defect in protein stability and/or heterodimer formation with MSH6 that affects chromatin localization.

### MSH2 missense mutants alter the DNA checkpoint response to alkylation damage

**Figure 3:**
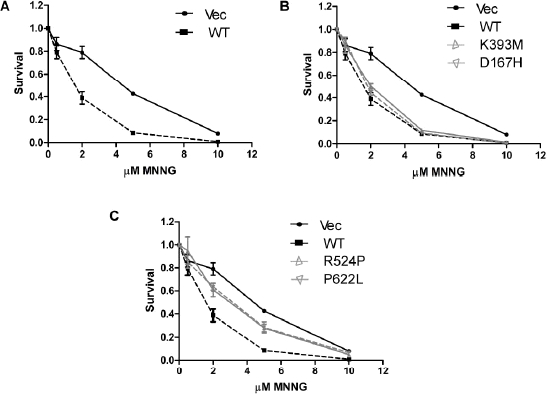
MSH2 missense mutations alter sensitivity to DNA alkylation damage. ( A) Response of wild-type and mutant MSH2 expressing cell lines treated with the indicated concentrations of MNNG was monitored by colony survival assays. Survival curves for wild-type MSH2 expressing and vector-only control cells displayed in all three panels for comparison purposes. (B) Survival curves for MSH2(D167H) and MSH2(K393M) expressing cells. (C) Survival curves for MSH2(R524P) and MSH2(P622L) expressing cells. Experiments were performed in quadruplicate.

An intact DNA MMR pathway is required for activation of cell cycle checkpoints and apoptosis in response to low-doses of MNNG (Stojic, et al., 2004b). Therefore, to test if missense mutations in *MSH2* could abrogate a normal DNA checkpoint response to MNNG we performed a colony survival assay. A clear difference in resistance was observed between the vector only cells and MSH2(WT) cells at lower concentrations of MNNG (Figure 3). Both MSH2(D167H) and MSH2(K393M) were able to restore sensitivity to MNNG similar to MSH2(WT), while MSH2(R524P) and MSH2(P622L) were significantly more resistant than MSH2(WT) (Figure 3). Furthermore, no significant difference in cell survival was observed for MSH2(R524P) and MSH2(P622L) compared to vector only cells.

**Figure 4:**
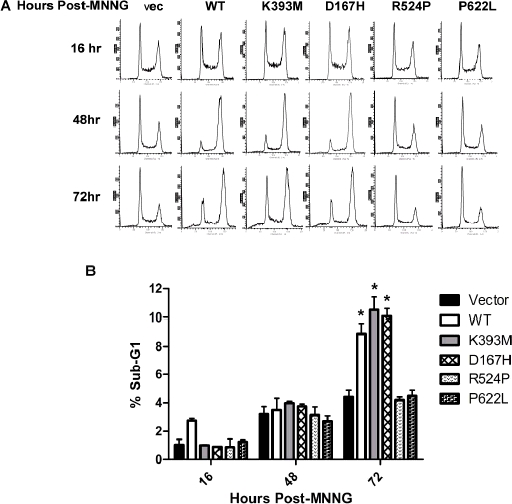
MSH2 missense mutants disrupt downstream responses to DNA alkylation damage. (A) Cell cycle progression of MNNG-treated wild type and mutant MSH2 expressing cells monitored by flow cytometry. (B) The sub-G_1_ population from cells at 72h post-treatment was quantified from two independent experiments. Asterisks indicate a p-value >0.05 by an unpaired t-test.

To further dissect the function of these missense mutations in response to alkylation damage we monitored the induction of a G_2_ cell cycle arrest. By 48 h post-treatment the majority of cells expressing MSH2(WT), MSH2(D167H), and MSH2(K393M) arrested in the G_2_/M phase of the cell cycle (Figure 4A). In general, vector only, MSH2(R524P), and MSH2(P622L)-expressing cells displayed unperturbed cell cycle profiles in response to MNNG (Figure 4A). It has been reported that following induction of a G_2_ arrest, MMR-proficient cells treated with MNNG undergo apoptosis (Schroering and Williams, 2008). At 72 h there was a significant increase in the population of sub-G_1_ cells in the MSH2(WT), MSH2(D167H) and MSH2(K393M) lines compared to vector only, MSH2(R524P), and MSH2(P622L) suggesting increased apoptosis (Figure 4B).

### MSH2 missense mutations abrogate or diminish DNA MMR activity *in vivo*

**Figure 5:**
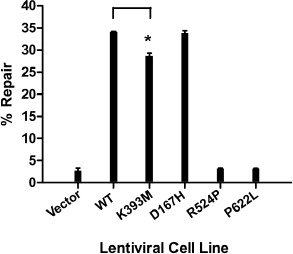
MSH2 missense mutations alter DNA mismatch repair activity *in vivo*. Mismatch repair was monitored in wild-type and mutant MSH2 expressing cell lines transfected with a heteroduplex plasmid encoding EGFP with a premature stop codon and RFP as a transfection control. Flow cytometry data from at least two independent experiments was quantitated. The asterisk represents a p-value > .001 by an unpaired t-test.

The repair capabilities of *MSH2* missense mutations have been previously characterized using complementation assays in bacteria and resistance plate assays in yeast (Ellison, et al., 2001; Gammie, et al., 2007; Lützen, et al., 2008; Martinez and Kolodner, 2010). Here, we have utilized an *in vivo* DNA mismatch repair assay in human cells (Zhou, et al., 2009). As expected, the vector only cell line was incapable of restoring EGFP fluorescence. By contrast, the number of EGFP positive MSH2(WT) cells increased greater than 10-fold (Figure 5). Both the MSH2(R524P) and MSH2(P622L) cell lines were unable to support MMR while the MSH2(D167H) expressing cells restored EGFP fluorescence similar to WT (Figure 5). MSH2(K393M) cells were appreciably more competent for repair than the vector only cells, though there was a slight, but significant decrease in repair compared to WT (Figure 5).

## DISCUSSION

To date the functional characterization of MSH2 missense mutations has been performed in model organisms such as bacteria and yeast or *in vitro* (Ellison, et al., 2001; Gammie, et al., 2007; Heinen, et al., 2002; Lützen, et al., 2008; Martinez and Kolodner, 2010; Ollila, et al., 2008; Ollila, et al., 2006). Characterization of LS-associated missense mutations in MSH2 using a human expression system is limited (Belvederesi, et al., 2008; Ollila, et al., 2006). Therefore, in our current study, we stably introduced four LS-associated missense mutations into the MSH2-null human endometrial cancer cell line Hec59 to determine their effects on *in vivo* cellular functions such as DNA repair and checkpoint response (Table 1).

**Table 1:** Summary of MSH2 missense variant functional analyses

Variant	Expression	Accumulation on chromatin	Protein interactions	Sensitivity to MNNG	Cell cycle arrest/apoptosis	Repair
D167H	+	+/−	+/−	+	+	+
K393M	+	+/−	+/−	+	+	+/−
R524P	+	−	−	−	−	−
P622L	+/−	−	−	−	−	−

(+) Proficient compared to WT MSH2

(−) Deficient compared to WT MSH2

(+/−) Reduced compared to WT MSH2

In our previous *in vitro* biochemical studies of these four missense mutants, we had observed a modest reduction in binding of MSH2(D167H) and MSH2(K393M) to a small oligonucleotide duplex containing a mismatch and a significant decrease in sliding clamp formation on a circular, mismatched DNA template (Heinen, et al., 2002). Consistent with these *in vitro* observations, we observed the accumulation of MSH2(D167H) and MSH2(K393M) on damaged chromatin to a slightly lesser extent compared to MSH2(WT). However, both mutants displayed increased sensitivity to DNA alkylation damage and increased repair over the vector only cells suggesting that the reduction in chromatin accumulation does not alter the DNA checkpoint response or repair efficiency. Interestingly, the level of repair observed in the MSH2(K393M) expressing cells was slightly reduced compared to the MSH2(WT) cells and may warrant further investigation.

In this study MSH2 protein expression levels were similar to endogenous expression observed in equivalent amounts of HeLa extracts (data not shown). However, the levels of mutant protein may still be higher than in tumors from an LS patient if they underwent a loss of heterozygosity (LOH) event and therefore express a hemizygous level of mutant protein. It will be interesting to test whether reduced expression of MSH2(D167H) or MSH2(K393M) would result in defective MMR function compared to a similarly reduced level of wild-type MSH2. Additionally, it would be interesting to examine the variants in combination with other MMR gene variants. Recently it has been reported that the combination of weak MMR alleles in *Saccharomyces cerevisiae* produces a strong polygenic defect in MMR functions and suggests an important additional layer of genetic complexity (Martinez and Kolodner, 2010). This study demonstrated that the combination of the *D164H* allele (analogous to D167H in our study) with an *msh6*Δ mutation resulted in a slight increase in mutation rate. Our data to this point, however, suggest that both variants are, at most, only very weakly contributing alleles.

The inability of MSH2(R524P) to accumulate on damaged chromatin is entirely consistent with our previous *in vitro* analyses showing a defect in mismatch recognition (Heinen, et al., 2002). However, MSH2(R524P) chromatin localization during S-phase was similar to WT protein in undamaged cells. This observation may reveal a damage-independent interaction between MSH2-MSH6 and chromatin that occurs normally during every S-phase. One possibility is that the MMR proteins are tethered to the newly synthesized chromatin during DNA replication to enhance the search for mismatches. Indeed this has been postulated as one of the consequences of MSH2-MSH6 interaction with PCNA (Lau and Kolodner, 2003), though an interaction between chromatin bound MSH2(R524P) and PCNA is barely detectable. Analysis of MSH2(P622L) expression revealed a ∼50% reduction with no detectable MSH6 and MSH3 protein, which taken together suggest a stability defect of the MSH2(P622L) protein which affects its ability to form a heterodimer with MSH6. These data are consistent with studies of the corresponding mutation in yeast Msh2 (P640L) (Gammie, et al., 2007). However, two *in vitro* studies have shown that MSH2(P622L) and MSH6 interact to form a stable heterodimer (Heinen, et al., 2002; Lützen, et al., 2008). This discrepancy demonstrates the value of examining these mutant proteins *in vivo* in human cells. Importantly, both MSH2(R524P) and MSH2(P622L) were repair-deficient similar to the vector only cells, which taken together with the inability of these proteins to elicit a DNA checkpoint in response to DNA alkylation damage is strong evidence that the R524P and P622L MSH2 variants are pathogenic.
